# Triglyceride-glucose indices predict all-cause mortality after stroke in NHANES 1999–2018

**DOI:** 10.3389/fnagi.2025.1617419

**Published:** 2025-05-30

**Authors:** Jiaqian Zheng, Weiwen Mao, Mengqian Sang, Xinyu Pan, Yiluo Xie, Yichi Xie

**Affiliations:** ^1^Department of Respiratory Medicine, Changzhou Geriatric Hospital Affiliated to Soochow University, Changzhou, China; ^2^Department of Respiratory Medicine, Changzhou No.7 People’s Hospital, Changzhou, China; ^3^Department of Clinical Medicine, Bengbu Medical University, Bengbu, China; ^4^Department of Radiology, The Affiliated Suzhou Hospital of Nanjing Medical University, Suzhou, China

**Keywords:** stroke, TyG-related index, NHANES, mortality, retrospective cohort analysis

## Abstract

**Objective:**

The present study explores the prognostic relevance of triglyceride-glucose–based indices in assessing post-stroke survival among affected individuals.

**Methods:**

This study utilized a multifaceted analytical approach to assess how triglyceride-glucose–based indicators relate to death risk in stroke patients. This study was analyzed using a multivariate Cox proportional risk regression model incorporating sampling weights, while a restricted cubic spline function was introduced to assess trends in non-linear associations between exposure variables and outcomes. In addition, interaction terms were set and stratified analyses were conducted to verify the robustness and heterogeneity of the model results.

**Results:**

This research ultimately included 796 individuals diagnosed with stroke. When adjusting for a wide range of potential confounders, those in the top TyG-BMI quartile exhibited the most pronounced reduction in mortality risk compared to individuals in the lowest category, with a hazard ratio of 0.20 (95% CI: 0.08–0.50), highlighting its protective potential across TyG-BMI. In contrast, individuals falling within the fourth quartile of the TyG-WHtR index demonstrated the strongest positive correlation with the risk of all-cause mortality (Hazard Ratio = 4.61, 95% CI: 1.77–12.00). Moreover, analysis using restricted cubic splines indicated a significant non-linear association between TyG-BMI levels and mortality outcomes (*p* < 0.05). No statistical interactions were observed between mortality outcomes and demographic or clinical variables including age, sex, smoking, asthma, coronary artery disease, diabetes, or hypertension across any TyG-related indices (*p* > 0.05).

**Conclusion:**

The study outcomes suggest that stroke patients with reduced TyG-BMI and elevated TyG-WHtR levels tend to face increased mortality risks. Nonetheless, addressing obesity may be crucial in exploring potential causal pathways.

## Introduction

The abrupt onset and quick progression of localized or generalized neurological impairments are the clinical hallmarks of stroke, an acute cerebrovascular illness (Mendelson and Prabhakaran, 2021). Globally, stroke represents a major contributor to mortality and disability, with its widespread occurrence and high fatality rate making it a persistent burden on healthcare systems. In many cases, it leads to prolonged impairment in daily functioning (Feigin and Owolabi, 2023). The incidence of stroke is expected to keep increasing as the population ages. Data from 2019 suggest that stroke affected nearly 12.2 million people worldwide, resulting in about 6.6 million deaths and leaving more than 101 million individuals with a prior stroke experience (GBD 2019 Stroke Collaborators, 2021). Acute stroke has been linked to a substantial decline in longevity, with average survival reduced by about 5.5 years relative to population norms (Peng et al., 2022). Considering the growing global burden posed by stroke, it is crucial for healthcare systems worldwide to rapidly recognize and manage key determinants associated with increased mortality.

Obesity is widely recognized as a major contributor to stroke risk, although the biological mechanisms involved are intricate and have yet to be fully clarified (Towfighi and Ovbiagele, 2008). Central obesity has been closely associated with elevated risks of multiple chronic conditions, notably type 2 diabetes, fatty liver disease unrelated to alcohol intake, and cerebrovascular complications (Lavie et al., 2009; Rhee, 2018; Vusirikala et al., 2020). While body mass index, waist-to-height proportion, and waist size are routinely applied in gauging obesity levels, these indicators fall short in accurately reflecting metabolic risk tied to fat accumulation, primarily because they fail to distinguish adipose tissue from lean muscle (Antonopoulos et al., 2016). Developed as a simplified surrogate for insulin resistance, the TyG index integrates triglyceride and fasting glucose concentrations, offering a convenient approach to metabolic risk evaluation (Simental-Mendía et al., 2008). As a simplified composite based on triglyceride and glucose concentrations, the TyG index has gained recognition for its utility in identifying insulin resistance in a low-cost and efficient manner (Tahapary et al., 2022). Incorporating anthropometric variables such as BMI, waist-to-height ratio, and waist circumference, TyG-related composite indices have demonstrated superior performance in estimating cardiovascular event likelihood (Dang et al., 2024) and related outcomes such as depression (Sun et al., 2025), hypertension (Zhao et al., 2025) and obstructive sleep apnea (Gong et al., 2025).

Accumulating evidence suggests that metrics based on triglyceride-glucose levels are closely linked to stroke risk in various populations. Previous studies have demonstrated that elevated TyG-WHtR levels are associated with an increased prevalence of stroke, highlighting the significance of managing blood glucose and lipid levels in stroke prevention (Xu et al., 2025). Long-term elevation of the TyG index among hypertensive individuals has been correlated with a heightened risk of overall and ischemic stroke (Huang et al., 2022). Studies have also found a significant association between higher TyG-BMI levels and an increased risk of stroke among middle-aged and elderly individuals in China (Shao et al., 2024). Despite growing interest in TyG-related metabolic markers, their prognostic significance in stroke-associated mortality remains underexplored. To date, no national cohort study has specifically evaluated the association between TyG-related indices and long-term mortality in stroke patients. This study draws upon nationally representative NHANES data to investigate how variations in TyG-based indices may influence fatal outcomes among stroke survivors, thereby addressing a critical knowledge gap and providing novel insights into metabolic predictors of post-stroke prognosis.

## Methods

### Sample source and design

**Figure 1:**
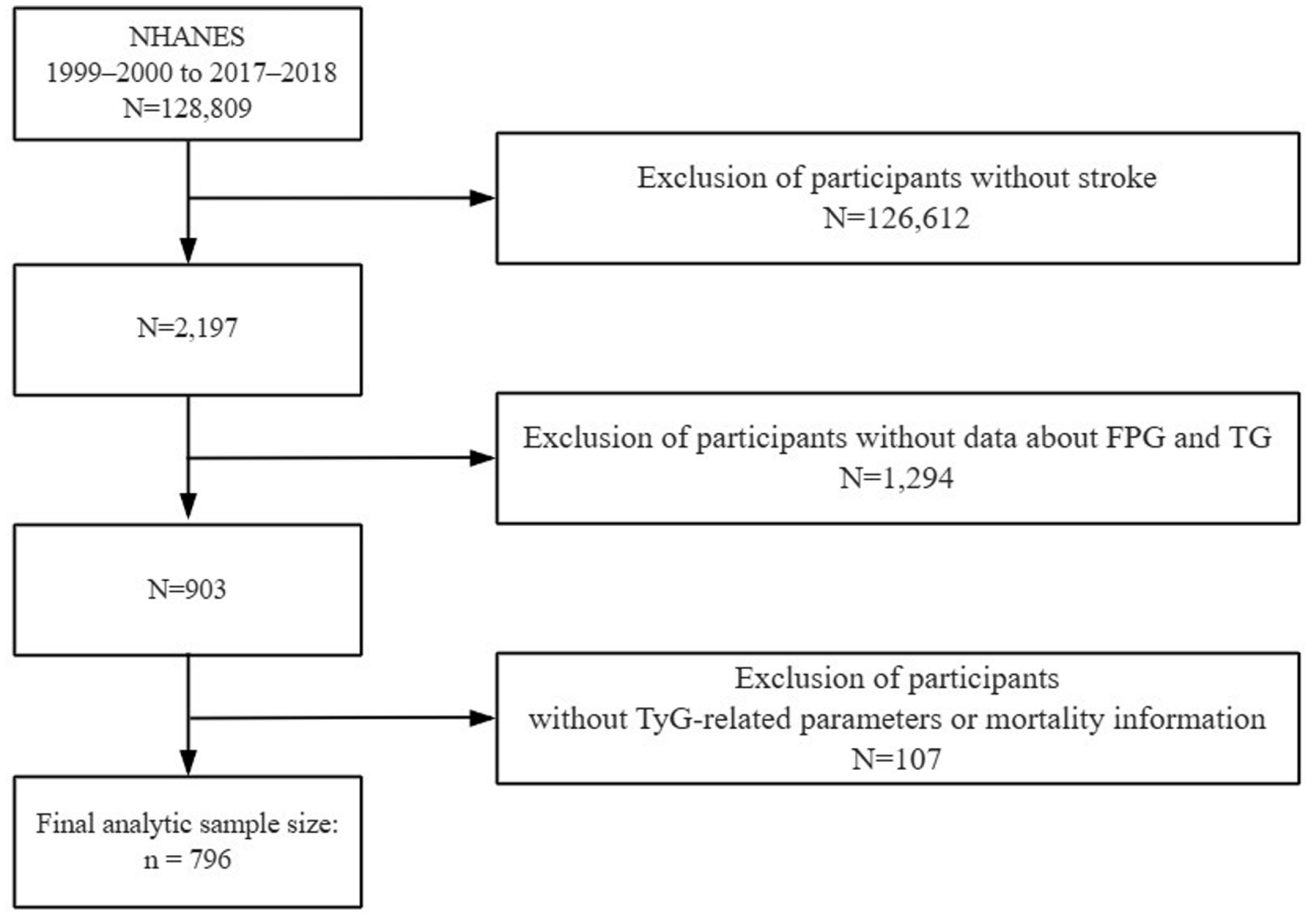
The flow diagram shows participant selection from NHANES 1999–2000 to 2017–2018, outlining inclusion and exclusion criteria.

To capture a representative snapshot of the U.S. non-institutionalized civilian population, the National Center for Health Statistics (NCHS) implemented a stratified, multistage, and probabilistic sampling strategy. Informed consent was obtained from all participants. The dataset was drawn from the National Health and Nutrition Examination Survey (NHANES), a cross-sectional national survey aimed at assessing nutritional conditions and public health risks. Details of the survey’s framework and public datasets can be accessed via the CDC’s official website^1^. Although NHANES data are collected cross-sectionally, this analysis is based on a prospective cohort design by linking baseline exposure variables with longitudinal mortality outcomes from the National Death Index. In this study, we analyzed data across 10 survey cycles from 1999 to 2018 to investigate how TyG-associated metrics relate to mortality in individuals with stroke. Exclusion criteria for the primary analyses included (1) not being a stroke patient or missing information on stroke; (2) missing data on fasting glucose and triglycerides; and (3) unavailability of information on all-cause mortality and missing information on other TyG-related indices. The final sample for this analysis was 796 individuals (Figure 1). It included 455 surviving participants and 341 deceased participants.

### Exposure and outcome

Laboratory data, including fasting triglyceride and glucose levels, were obtained from the NHANES database. All blood samples were collected after participants had fasted for at least 8 h, in accordance with NHANES standard protocols. Insulin resistance can be assessed by combining fasting blood glucose levels with triglyceride levels, a method represented by the Triglyceride Glucose (TyG) index. Participants underwent initial biochemical testing, during which venous blood was drawn to evaluate fasting glucose concentrations and lipid content, including triglycerides (Yan et al., 2022; Ding et al., 2024). The triglyceride-glucose index (TyG) was derived using the natural logarithm of the product of fasting glucose and triglyceride concentrations divided by two: TyG = ln [(fasting glucose in mg/dL × triglycerides in mg/dL)/2]. To enhance its clinical relevance, this index was further combined with anthropometric indicators of obesity, including body mass index (BMI), waist circumference (WC), and waist-to-height ratio (WHtR), forming a set of composite metabolic indicators (Ashwell et al., 2012). Body mass index (BMI) is derived by taking a person’s weight and dividing it by the square of their height. In contrast, waist-to-height ratio (WHtR) reflects the proportion between waist circumference and body height. The specific calculation methods for each index are outlined below (Simental-Mendía et al., 2008; Zheng et al., 2016; Lim et al., 2019; Korhonen et al., 2021):

- TyG-WHtR = TyG × WHtR.
- TyG-WC = TyG × Waist Circumference (cm).
- TyG-BMI = TyG × BMI (kg/m^2^).

Based on the distribution of TyG-associated indices, subjects were stratified into four quartiles (Q1–Q4), with the lowest quartile acting as the reference point. Stroke diagnosis was established through participant self-disclosure during standardized interviews, which followed a validated medical condition assessment protocol involving clinician-confirmed reports (Hu et al., 2023; Mao et al., 2024). Respondents answering “yes” were classified as having experienced a stroke. Mortality data were acquired by merging NHANES datasets with publicly available records from the National Death Index (NDI), with follow-up data extending through December 31, 2019. Cause-of-death information was derived from official death certificates and categorized according to ICD-10 coding standards. Deaths from any cause were considered under the definition of all-cause mortality.

### Covariate assessment

Expanding upon earlier evidence, this analysis adjusted for numerous confounding factors to more accurately evaluate how TyG-based indicators relate to mortality among those diagnosed with stroke. These include demographic data, physical examination findings, behavioral risk factors, chronic non-communicable diseases, and laboratory testing results (Jiang et al., 2024; Liang et al., 2025; Xu et al., 2025). Collected demographic characteristics encompassed sex (male or female), chronological age, ethnic background (including Mexican American, other Hispanic groups, non-Hispanic white, non-Hispanic black, and others), educational attainment (ranging from high school and below to college and higher), and household economic status measured by the poverty income ratio (PIR). Physical examination indicators related to body measurements encompassed BMI, waist circumference, and standing height. Lifestyle-related risk variables involved smoking behavior—defined as a lifetime history of consuming 100 or more cigarettes (yes/no)—and average alcohol intake over the past year. Information on participants’ past medical conditions was collected, focusing on prevalent non-infectious chronic diseases including diabetes, high blood pressure, asthma, and ischemic heart disease. Additionally, laboratory data included measurements of LDL-C concentrations.

### Statistical analysis

All statistical procedures adhered to the analytical guidelines set by the CDC, incorporating adjustments for the NHANES dataset’s multistage and stratified sampling design. Survey weights provided by NHANES were applied throughout the analysis process to ensure population representativeness, including corrections for over-sampling of minority subgroups. Results were deemed statistically significant at a threshold of *p* < 0.05. To minimize potential bias, this study used medians or means to interpolate missing covariates.

To compare baseline distributions among TyG quartile classifications, appropriate statistical tests were applied—ANOVA for numerical indicators and chi-square analysis for categorical measures. Given the asymmetrical distribution of the TyG index, a natural log transformation was performed prior to its inclusion in the Cox proportional hazards model. To assess survival variation among subgroups, Kaplan–Meier estimations were plotted, and statistical significance across groups was determined using the log-rank method. The impact of TyG levels on stroke-related mortality was quantified through Cox survival modeling, with outcomes expressed as HRs and corresponding 95% CIs. To mitigate confounding and systematically explore the TyG–mortality association, three progressively adjusted models were constructed: Model 1 was unadjusted; Model 2 incorporated demographic and socioeconomic factors (age, sex, ethnicity, education, PIR); and Model 3 further controlled for anthropometric, clinical, and behavioral covariates including BMI, height, waist size, LDL-C, tobacco and alcohol use, as well as comorbidities (e.g., hypertension, diabetes, CHD, asthma). Restricted cubic spline (RCS) regression was employed to assess possible non-linear relationships between variables. If the association showed non-linearity after controlling for all pertinent factors, threshold probabilities were calculated, and a threshold effects analysis model was used to examine associations on each side of the threshold.

To further validate the robustness of the findings, we conducted interaction assessments along with stratified subgroup evaluations. The subgroup variables considered in this analysis included age group (≤65 and >65 years), sex, smoking habits, and whether participants had conditions such as asthma, coronary heart disease, hypertension, or diabetes. Data analysis and statistical processing were performed using the survey, tidyverse, and pROC packages in R (version 4.3.2), as well as EmpowerStats (version 4.2). Statistical significance was determined at a two-sided *p*-value threshold of less than 0.05.

## Results

### Demographic and clinical characteristics of stroke patients

Supplementary Table S1 summarizes participant profiles stratified by quartiles of TyG and its derived indices, including TyG-BMI, TyG-WC, and TyG-WHtR. Individuals with hypertension and educational attainment at or below the high school level made up a large proportion of those in the upper TyG-WHtR quartile. In addition, a gradual increase in the proportions of male and diabetic participants was observed with rising TyG-WHtR levels. Comparable distribution patterns were also noted at baseline across TyG, TyG-WC, and TyG-BMI quartiles.

The baseline demographic and clinical profiles of individuals with stroke are summarized in Table 1, distinguishing between those who survived and those who died from all causes prior to the study endpoint. On average, participants who did not survive had an age of 71.13 years, were predominantly non-Hispanic white, tended to have completed no more than a high school education, and generally reported lower household income relative to the poverty threshold (PIR). They had a slightly elevated TyG index compared with participants who did not die, and a lower TyG-BMI, lower prevalence of asthma, and lower prevalence of coronary heart disease.

**Table 1:** Demographic characteristics of individuals with stroke according to status, weighted for representativeness.

Characteristic	Assumed alive (*n* = 455)	Assumed deceased (*n* = 341)	*p*-value
Age (yrs), mean ± sd	59.85 (58.25,61.45)	71.13 (69.42,72.83)	<0.001
PIR, mean ± sd	2.52 (2.31,2.73)	2.18 (2.00,2.37)	0.017
Drinking, mean ± sd	2.30 (2.08,2.51)	2.13 (2.00,2.26)	0.188
LDL-cholesterol (mmol/L), mean ± sd	109.45 (104.98,113.92)	109.20 (104.12,114.27)	0.936
TyG, mean ± sd	9.47 (9.39,9.56)	9.60 (9.52,9.68)	0.046
TyG-BMI, mean ± sd	293.90 (283.87,303.92)	276.39 (265.71,287.08)	0.018
TyG-WC, mean ± sd	994.92 (968.32,1021.51)	990.02 (961.34,1018.71)	0.807
TyG-WHtR, mean ± sd	6.01 (5.86,6.16)	5.98 (5.82,6.15)	0.806
Gender, *N* % (95% CI)			0.064
Male	38.43 (31.89,45.42)	47.60 (41.04,54.24)	
Female	61.57 (54.58,68.11)	52.40 (45.76,58.96)	
Race, *N* % (95% CI)			0.003
Mexican American	4.91 (3.41,7.03)	3.31 (2.02,5.36)	
Other Hispanic	3.72 (2.40,5.73)	2.27 (0.80,6.31)	
Non-Hispanic White	64.85 (59.14,70.16)	79.17 (73.45,83.92)	
Non-Hispanic Black	16.48 (13.42,20.08)	11.13 (8.42,14.57)	
Others	10.04 (6.29,15.64)	4.12 (1.99,8.35)	
Education, *N* % (95% CI)			0.024
≤High school	53.78 (46.74,60.68)	66.42 (59.98,72.31)	
College	28.57 (23.49,34.26)	20.12 (15.17,26.19)	
>College	17.64 (12.62,24.12)	13.45 (9.54,18.65)	
Smoking, *N* % (95% CI)			0.326
Yes	61.48 (56.13,66.55)	65.51 (58.23,72.13)	
No	38.52 (33.45,43.87)	34.49 (27.87,41.77)	
Diabetes, *N* % (95% CI)			0.618
Yes	26.39 (21.57,31.85)	28.39 (22.76,34.77)	
No	73.61 (68.15,78.43)	71.61 (65.23,77.24)	
Hypertension, *N* % (95% CI)			0.059
Yes	67.30 (60.65,73.33)	75.66 (68.97,81.30)	
No	32.70 (26.67,39.35)	24.34 (18.70,31.03)	
Asthma, *N* % (95% CI)			0.004
Yes	27.99 (22.50,34.24)	14.74 (10.15,20.94)	
No	72.01 (65.76,77.50)	85.26 (79.06,89.85)	
Coronary heart disease, *N* % (95% CI)			0.010
Yes	13.97 (10.22,18.83)	23.39 (18.11,29.66)	
No	86.03 (81.17,89.78)	76.61 (70.34,81.89)	

### Evaluating the prognostic value of TyG-based indicators in stroke-related mortality

**Figure 2:**
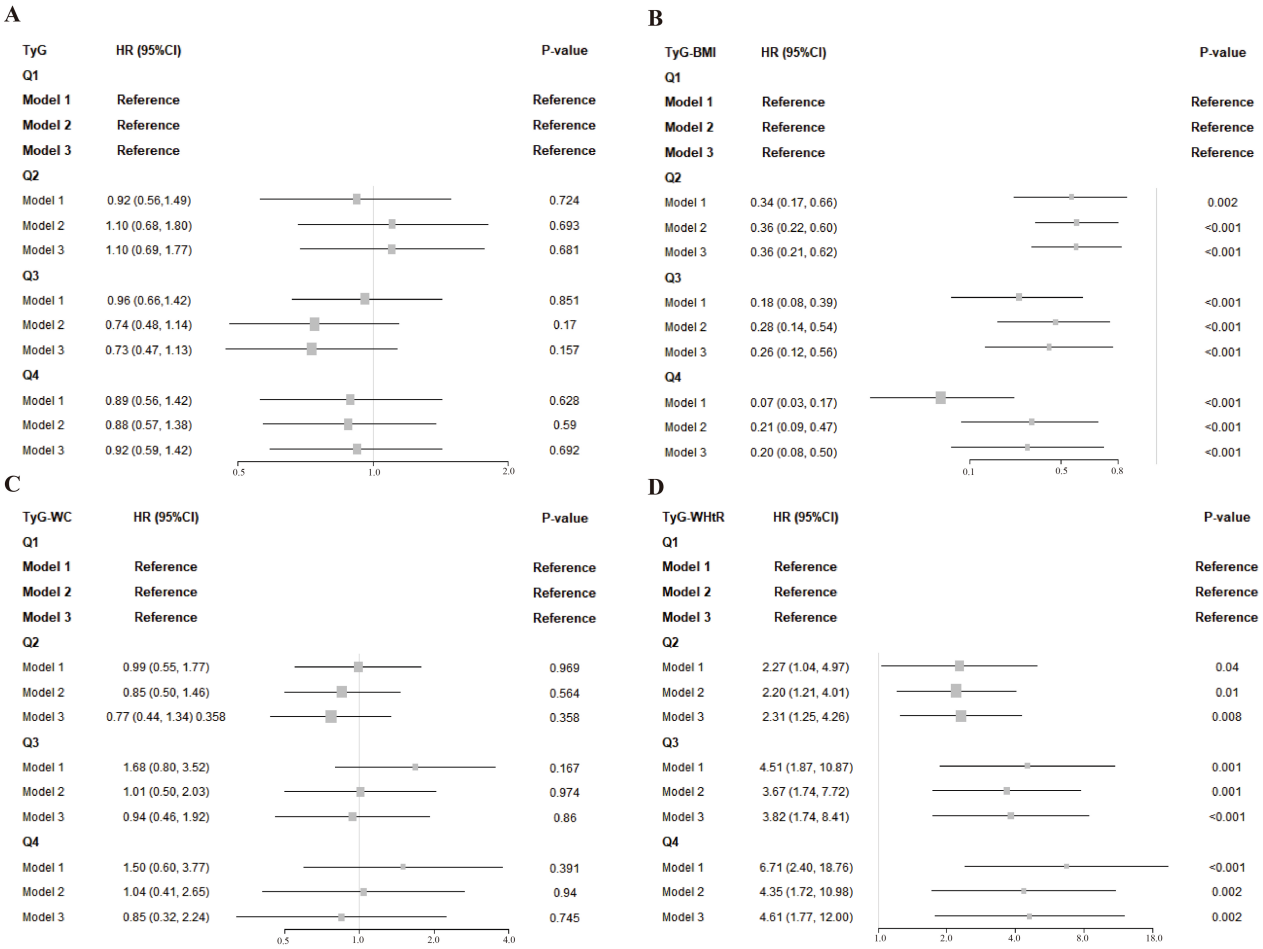
**(A–D)** Associations of TyG and its composite indices with post-stroke mortality across different models. Model 1: This model did not include any adjustments. Model 2: Adjustments were made for age, race, gender, education level, and family PIR. Model 3: Further adjustments based on 2a Model 2 included LDL-cholesterol, drinking status, smoking status, hypertension, diabetes, asthma and coronary heart disease.

Figure 2 illustrates the relationships between TyG-derived metrics and overall mortality risk in stroke patients, reporting hazard ratios (HRs) alongside their 95% confidence intervals (CIs). Utilizing regression models with survey weights and adjusting for multiple covariates, we treated TyG and its related indices as standardized continuous variables (per standard deviation). After adjusting for relevant factors, we developed three distinct models and conducted Cox regression analysis.

Taking the lowest quartile (Q1) as the comparison baseline, TyG and its related indices were categorized into four levels, which revealed distinct trends in relation to overall mortality. Specifically, TyG-BMI in Q2 (HR = 0.36, 95% CI: 0.21–0.62), Q3 (HR = 0.26, 95% CI: 0.12–0.56), and Q4 (HR = 0.20, 95% CI: 0.08–0.50) showed significant inverse relationships with mortality risk, implying that higher TyG-BMI values might be associated with improved survival in stroke patients. Adjusted analysis revealed that those with the highest TyG-BMI values had a markedly decreased likelihood of death— 80% lower—when contrasted with individuals in the lowest measurement group. In contrast, the TyG-WHtR index exhibited a significant positive association with all-cause mortality across higher quartiles: Q2 (HR = 2.31, 95% CI: 1.25–4.26), Q3 (HR = 3.82, 95% CI: 1.74–8.41), and Q4 (HR = 4.61, 95% CI: 1.77–12.00), relative to Q1. A clear upward trend in mortality risk was observed with rising TyG-WHtR levels, with fully adjusted models indicating that individuals in the highest quartile faced a 3.61-fold elevation in death risk compared to those in the reference group.

### Curvilinear associations between TyG-derived metrics and risk of all-cause mortality

**Figure 3:**
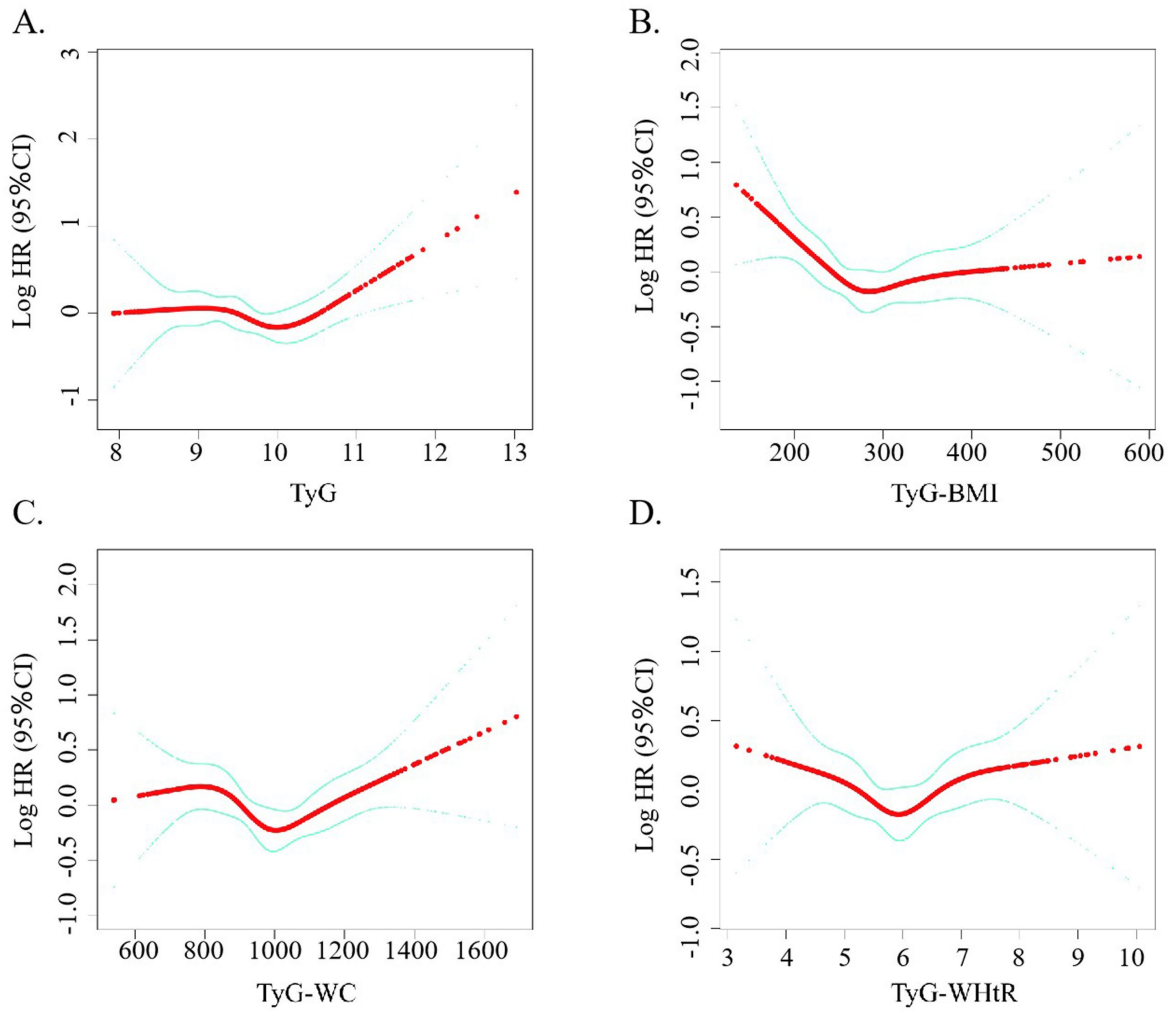
**(A–D)** Restricted cubic spline curve for the association between TyG-related indicators and the mortality rate of stroke patients.

As visualized in Figure 3, a non-linear dose-dependent trend was detected between the TyG-based indicators and total mortality. Notably, the TyG-BMI metric followed an L-shaped trajectory, with a critical inflection at 248.18. Statistical evaluation using the log-likelihood ratio confirmed the significance of this curvilinear relationship, as all *p*-values were under the 0.05 threshold.

As presented in Table 2, TyG-BMI values falling below the identified threshold were significantly inversely related to all-cause mortality among stroke patients (*p* = 0.001), whereas values exceeding that threshold showed a significant positive association (*p* = 0.005). Although initial findings suggested a possible non-linear trend between TyG-WC and mortality, additional investigation is necessary to validate this relationship. On the other hand, no statistically meaningful association was observed for TyG-WHtR in any of the models tested.

**Table 2:** Analysis of the threshold effect of TyG-related indicators on all-cause mortality in stroke.

All-cause mortality	TyG	TyG-BMI	TyG-WC	TyG-WHtR
	HR (95% CI), *p*			
Inflection point	10.292	248.18	984.02	5.69
Index < inflection point	0.88 (0.70, 1.10) 0.262	0.99 (0.99, 1.00) 0.001	1.00 (1.00, 1.00) 0.034	0.77 (0.58, 1.01) 0.063
Index > inflection point	1.76 (1.18, 2.61) 0.005	1.00 (1.00, 1.00) 0.546	1.00 (1.00, 1.01) 0.023	1.17 (0.99, 1.38) 0.069
Log-likelihood ratio	0.011	0.005	0.009	0.031

### Subgroup-based evaluation of the relationship between TyG-derived indicators and all-cause mortality

**Figure 4:**
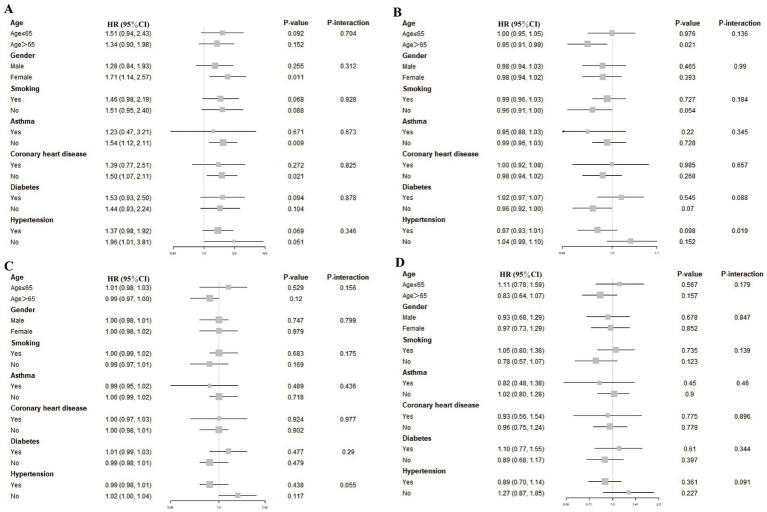
Associations between TyG-based indices and stroke-related mortality across various subgroups. To assess the associations between stroke-related mortality and the four TyG-derived metrics—TyG **(A)**, TyG-BMI **(B)**, TyG-WC **(C)**, and TyG-WHtR **(D)**—stratified subgroup evaluations were performed. These models were adjusted for a comprehensive set of covariates, including demographic factors (age, sex, race, education, income level), physical measures (BMI, height, waist size, LDL-C), lifestyle behaviors (smoking and alcohol use), and chronic conditions (hypertension, diabetes, coronary disease, and asthma).

Figure 4 displays subgroup analysis results, accounting for relevant confounding variables such as age, sex, smoking status, asthma, coronary artery disease, diabetes, and hypertension. In general, the correlations between TyG-associated indices and overall mortality did not reach statistical significance across the majority of examined subgroups. Notably, a statistically significant association was observed between the TyG index and elevated mortality among female stroke patients (HR: 1.71, 95% CI: 1.14–2.57), as well as those stroke without a history of asthma (HR: 1.54, 95% CI: 1.12–2.11). Among participants aged over 65, higher TyG-BMI values were associated with a reduced risk of death (HR: 0.95, 95% CI: 0.91–0.99), indicating a potentially protective effect. No significant interaction was detected across stratifications by demographic or clinical characteristics (all *p*-values exceeded 0.05), suggesting that the relationships observed were generally consistent across subpopulations.

## Discussion

As far as we are aware, this study is the first to assess the association between TyG-related markers and overall mortality outcomes in stroke patients within a U.S.-based population. The study comprised 796 participants representative of the national population. The analysis indicated a negative correlation between TyG-BMI and risk of death, while TyG-WHtR demonstrated a positive association. These patterns persisted even after adjusting for an extensive array of potential confounders, such as demographic characteristics, socioeconomic status, metabolic indicators, and cardiovascular risk factors—including age, sex, ethnicity, education, income, LDL cholesterol, lifestyle behaviors, and existing conditions like hypertension, diabetes, asthma, and coronary artery disease. Specifically, participants falling into the highest quartile of TyG-WHtR exhibited a 4.61 times greater risk of death, whereas those in the uppermost TyG-BMI quartile experienced a 20% lower likelihood of mortality.

There are two probable explanations for this outcome. The first is that we found that most of the patients in this study cohort were overweight. Contrary to conventional understanding, some evidence points to better post-stroke prognoses among individuals with higher body weight—a pattern referred to as the “obesity paradox,” which challenges the typical associations found in general populations (Oesch et al., 2017; Xu et al., 2019). Evidence from CNSR II suggests that elevated body mass among ischemic stroke patients may be linked to enhanced survival, with overweight and obese individuals showing better prognoses than those at lower weight classifications (Hou et al., 2021). Second, both elevated and decreased glucose and lipid levels can be detrimental to health and exacerbate existing conditions. At low glucose levels, hyperinsulinemia leads to a stronger counter-regulatory response. This physiological response involves an increase in circulating stress hormones, including epinephrine and norepinephrine, which in turn promote vascular constriction and enhance platelet activity, ultimately heightening the likelihood of cardiovascular complications (Laitinen et al., 2003). In our study, we observed an apparent discrepancy between the categorical analysis (Figure 2D) and the restricted cubic spline (RCS) plot (Figure 3D) in describing the association between TyG-WHtR and all-cause mortality. While the categorical analysis showed a monotonic increase in hazard ratios across TyG-WHtR quartiles, the RCS model revealed a U-shaped relationship. This difference may be due to the limitations of the quartile-based analysis, which classifies continuous variables into broad categories and may not capture non-linear trends effectively. Specifically, the lowest risk zone identified in the RCS plot may fall between Q2 and Q3, but when grouped, both Q2 and Q3 may exhibit elevated risk compared to Q1. As a result, the U-shaped association is masked in the grouped analysis. Nevertheless, no noteworthy correlations between the stratified analyses were discovered in the subgroup analysis. The findings suggest variability in the relationship between different TyG values, their associated indices, and all-cause mortality risk among stroke patients. Consequently, TyG-WHtR and TyG-BMI may function as a cost-effective and clinically useful marker for early detection and risk prevention in stroke prognosis. This may be attributed to their ability to account for both metabolic dysfunction and obesity-related risk in a more comprehensive manner (Dang et al., 2024). BMI and WHtR may better reflect total or proportional adiposity, whereas WC alone may be influenced by abdominal shape and posture, potentially leading to measurement bias (Stefan et al., 2017). Additionally, WHtR has been proposed as a more stable predictor across different ethnicities and age groups. Previous studies have also suggested that TyG-WHtR is superior to WC in predicting cardiovascular and all-cause mortality due to its normalization for height and better reflection of visceral fat distribution (Ashwell et al., 2012).

Research involving both ischemic and hemorrhagic stroke indicates that diabetes mellitus has an average combined prevalence of 28%. Moreover, the coexistence of acute hyperglycemia and diabetes has been linked to poorer prognoses in individuals who have suffered a stroke (Lau et al., 2019), which agrees with what we found. Meta-analytic evidence suggests that diabetes independently contributes to elevated stroke mortality, regardless of patients’ baseline health profiles or other potential fatal factors. Preventing excess weight gain may help interrupt the advancement from prediabetic states to diabetes, and potentially reduce the probability of future stroke events (Zeitler and Piccini, 2021). In patients with type 2 diabetes, GLP-1 receptor agonists have been associated with lower incidence rates of stroke onset, cardiovascular events, and mortality linked to heart-related conditions (Malhotra et al., 2020). The findings from these studies indicate a potential link between diabetes and increased risk of death following stroke, though the underlying biological mechanism has yet to be fully elucidated.

Type 2 diabetes is commonly associated with impaired insulin effectiveness at the cellular level, a condition known as insulin resistance. The triglyceride-glucose index (TyG), among several indirect evaluation approaches, has emerged as a practical and validated indicator for quantifying this metabolic dysfunction (Defronzo, 2009; Tao et al., 2022). Insulin resistance (IR) is increasingly recognized as a possible shared mechanism underlying elevated stroke susceptibility. Research has shown that certain recently developed pharmacological agents can effectively lower IR levels and may contribute to reducing the likelihood of stroke occurrence (Kernan et al., 2002). Stroke can be categorized into two major forms: hemorrhagic and ischemic. Among them, ischemic stroke (IS) is the predominant type, representing more than 87% of stroke cases globally (Saini et al., 2021). Acute arterial occlusion is the main cause of IS, and may develop from thromboembolism due to atherosclerosis of the large arteries, cardiac embolism, small-vessel disease, and unidentified factors (Huang et al., 2024). IR increases platelet aggregation, hinders endothelial function, and encourages the production of thrombi. Furthermore, it interferes with lipid regulatory processes, promoting the progression of atherosclerosis and the accumulation of arterial plaques, which together facilitate the onset of ischemic stroke (IS) (Ding et al., 2022). Despite being the benchmark technique for insulin resistance evaluation, the hyperinsulinemic clamp is rarely used in routine practice due to its resource-intensive nature—requiring significant time, cost, and technical labor (Tahapary et al., 2022). The TyG index, frequently employed as an alternative indicator of insulin resistance, has also been associated with an increased likelihood of initial stroke events in individuals with no previous stroke history (Jiang et al., 2024). When supplemented with anthropometric indicators, the TyG index demonstrates improved capacity for characterizing fat distribution and identifying obesity-associated health risks, outperforming its standalone application (Er et al., 2016). TyG-BMI, TyG-WC, and TyG-WHtR are extended indices that combine TyG with different measures of obesity. Specifically, TyG-BMI integrates general obesity, TyG-WC captures central obesity, and TyG-WHtR reflects the body fat distribution relative to height. Retrospective evidence highlights differential links between TyG-BMI and TyG-WC and both acute clinical presentation and early-stage recovery in individuals undergoing their first ischemic cerebrovascular episode (Yu et al., 2024). Research conducted by Fu et al. reported individuals with obesity who had higher TyG-WHtR levels exhibited an elevated predisposition to stroke, suggesting this index may serve as a potential indicator of cerebrovascular vulnerability (Fu et al., 2025). Based on these studies, monitoring changes in these markers could be valuable for assessing metabolic function, potentially helping stroke patients achieve better prognoses.

This research investigates the link between TyG and its associated indicators and all-cause mortality among U.S. adults diagnosed with stroke. Drawing upon data from the National Health and Nutrition Examination Survey (NHANES), the study offers meaningful clinical evidence to support improvements in stroke outcome prediction. We performed comprehensive adjustments for numerous potential confounding variables, including demographic characteristics, physical and laboratory examination data, lifestyle factors, and chronic diseases. To ensure the reliability of the observed associations, additional subgroup analyses were carried out within defined populations. Moreover, sample weights were applied to ensure national representativeness and broader applicability. Overall, the TyG-related indicators appear to be accessible and cost-efficient tools for mortality risk stratification among stroke patients.

This study has several limitations that should be acknowledged. First, as a retrospective cohort study, the possibility of residual confounding and potential misclassification of exposure or outcomes cannot be entirely excluded, which may limit the ability to draw definitive causal inferences between TyG-related indices and mortality outcomes in stroke patients. Second, stroke diagnoses were based on self-reported data, which may be subject to recall bias or misclassification due to participants’ misunderstanding, limited health literacy, or memory lapses. Third, the analysis is susceptible to survivor bias, as it included only community-dwelling individuals who were alive at the time of baseline assessment, thereby potentially excluding individuals with more severe illness or early mortality. Fourth, exposure variables such as the TyG index were measured only once at baseline, which may not capture long-term metabolic fluctuations or temporal variability, limiting the capacity to evaluate dynamic exposure-outcome associations. Fifth, missing covariate data were imputed using median values or mean estimates methods, which, while practical, may underestimate the variance and ignore potential relationships between variables, thereby introducing bias into the regression estimates. Finally, because NHANES data are predominantly derived from U.S. adults, the generalizability of our findings to other populations may be limited. Differences in genetics, lifestyle factors, and environmental exposures across global populations may affect the applicability of these results outside the U.S. context.

## Conclusion

This analysis highlights that among stroke patients, a combination of low TyG-BMI and high TyG-WHtR levels corresponds with an increased likelihood of mortality from all causes. These composite indices may offer predictive value for identifying at-risk populations and guiding preventive clinical practices. To further evaluate these findings and offer insightful information for clinical treatment, prospective clinical trials are necessary.

## Data Availability

The original contributions presented in the study are included in the article/Supplementary material, further inquiries can be directed to the corresponding authors.

## Footnotes

^1^ https://www.cdc.gov/nchs/nhanes/
